# Biologism in Psychiatry: A Young Man’s Experience of Being Diagnosed with “Pediatric Bipolar Disorder”

**DOI:** 10.3390/jcm3020334

**Published:** 2014-03-28

**Authors:** Peter Parry

**Affiliations:** 1 Department of Psychiatry, University of Queensland, Herston, Brisbane, QLD 4006, Australia; E-Mail: p.parry1@uq.edu.au; Tel.: +61-7-3146-2422; Fax: +61-7-3146-2420; 2 Department of Psychiatry, Flinders University, Bedford Park, Adelaide, SA 5042, Australia

**Keywords:** bipolar disorder, childhood, adolescence, psychiatric diagnosis, bioethics, pediatrics, medical sociology, iatrogenic disease, consumer participation, polypharmacy

## Abstract

Pediatric bipolar disorder is a diagnosis that arose in the mid 1990s in the USA and has mostly remained confined to that nation. In this article a young American man (under a pseudonym) describes his experience of having the diagnosis throughout his adolescent years. His story was conveyed via correspondence and a meeting with the author, an Australian child psychiatrist. The young American’s story reveals several issues that afflict contemporary psychiatry, particularly in the USA, where social and economic factors have contributed to the rise of a dominant biomedical paradigm—or “biologism”. This focus on the “bio” to the relative exclusion of the “psychosocial” in both diagnosis and treatment can have serious consequences as this young man’s story attests. The author explores aspects of his tale to analyze how the pediatric bipolar disorder “epidemic” arose and became emblematic of a dominant biologism. This narrative points to the need, depending on the service and country, to return to or retain/improve a balanced biopsychosocial perspective in child and adolescent mental health. Child psychiatry needs to advocate for health systems that support deeper listening to our patients. Then we can explore with them the full range of contextual factors that contribute to symptoms of individual and family distress.

## 1. Introduction

This article arises from a dialogue between myself, an Australian child and adolescent psychiatrist, and a young American man about his experience of psychiatric treatment over the course of his adolescent years. But in a way it starts much earlier with an article I read during psychiatry training in 1990. The article in the *Australian and New Zealand Journal of Psychiatry* by Australian professor Derek Silove left a distinct impression on me [1]. Silove reported:

“A recent study visit to North America impressed on me the seriousness with which Australian psychiatry should consider the recent ideological shift in the USA to an extreme biological model of mental disorders. … In its most doctrinaire form, this monotheistic biologism rejects (or worse still, pays condescending lip service to) the roles of social, cultural and psychological factors in the genesis and treatment of psychiatric disorders and relegates mentalistic notions to the epiphenomenal waste heap.”

Sixteen years later I was reminded of Silove’s article when child psychiatric colleagues in Australia became aware of a controversial new diagnosis, “Pediatric Bipolar Disorder” (PBD), emanating from the USA. The death of a 4-year old girl in Boston on 3 psychotropics for PBD further highlighted the controversy. Colleagues and I critiqued a guest editorial favourable to PBD [2] that was published in the *Australian and New Zealand Journal of Psychiatry* [3], published an article on PBD “a controversy from America” [4] and conducted a survey of Australian and New Zealand child & adolescent psychiatrists on this issue [5].

My interest in PBD has led me to meet many excellent U.S. child psychiatry colleagues. Most hold views similar to the systemic biopsychosocial perspective I acquired in my training and practice of the profession here in Australia. They too express deep concern about the PBD “epidemic” in their nation. This article is not aimed at disparaging U.S. psychiatry. Nor is it to discount the true cases of bipolar disorder in young people that we see. However, I, like my American friends and other international colleagues, am motivated by a desire to see our field retain a balanced perspective. The PBD diagnostic epidemic is emblematic of the pressures and problems besetting the field. The DSM-5 [6] introduced a new diagnosis, Disruptive Mood Dysregulation Disorder (DMDD) specifically to curb the overdiagnosis of bipolar disorder in children and adolescents in the USA.

Because the USA leads many global trends, the PBD epidemic offers valuable lessons to global psychiatry and mental health care. Diagnostic upcoding factors—financial, social and bureaucratic pressures that foster increased use of particular diagnoses—are an international phenomenon. PBD and DMDD have their corollary in Australia, where an epidemic of Autistic Spectrum Disorder (ASD) relates to diagnostic upcoding factors embedded in educational and welfare benefits for children and families and health insurance rebates to health providers who diagnose ASD [7]. PBD did not receive sustained academic support in Australia or New Zealand and thus overdiagnosis of ASD seems to have played a similar role, though with much less accompanying medication.

### Dialogue with a Young American Man Recovering from a Diagnosis of PBD

In 2008, I posted some thoughts on a mental health website forum discussion of PBD. A 20 years old young American man wrote eloquently on the forum of his personal experience. We corresponded by email and in a 2013 study trip of my own to the USA I met with him and heard his story face to face.

The young man, whom I shall call “Adam” (not his real name), is now in his mid 20th. His verbal recollections were virtually word for word what he’d reported in the emails 5 years earlier. He is doing well in his university studies, is widely read and very knowledgeable about psychiatry and health related politics. He has had no psychiatric diagnosis, nor any psychotropic medication, since leaving home in 2008. However he still struggles with the iatrogenic trauma of the diagnosis in his life. He recalled “about 30 hospital admissions” during the period of the PBD diagnosis. He was continuing to benefit from psychotherapy and apart from a sense of profound regret for a lost adolescence, he’d had no symptoms that would meet criteria for an “Axis I” psychiatric disorder in the past 5 years.

Adam’s narrative is his subjective experience, and thus reliant on memory. However, he did show me several discharge summaries of his hospital admissions that corroborated his story. The documents included concern that Adam was suffering a degenerative neurological disorder at a time he was on multiple psychotropic medications but apparently without consideration of the cognitive impairing effects of the pharmacotherapy.

I shall now let “Adam” speak for himself, having only edited his emailed story for de-identification and to reduce repetition. The discussion will focus on the issues this articulate young man’s account raises.

## 2. Adam’s Story (From 2008/9 Email Exchange)

I don’t mind sharing most anything about how my extensive psychiatric contact has affected me. I’m almost 21 now. I was 12 when first diagnosed. I had suffered depression and anxiety including severe OCD, which has since disappeared. It should also be mentioned I come from a screwed-up family and was physically abused by a sibling. Parents divorced young. My mother had a lot of issues, *etc.* So it goes without saying there was a lot the psychiatrist should have asked if he was ever so inclined. But unfortunately, he holds a faculty appointment at (edited—A PBD oriented child and adolescent psychiatry clinic).

Within about three months, I was on 8 different medications at one time. Very scientific treatment—all the best—several anticonvulsants, several antipsychotics, a couple of antidepressants and lithium too.

Things got so bad, that I ended up being referred to the neurology department, for different opinions about strange symptoms I began having on this cocktail. Which resulted in their giving me a working diagnosis of some kind of mitochondrial myopathy. “Bipolar plus mitochondrial disease” as it went. Which I have been told only recently could have been precipitated by the huge amounts of divalproex I was taking. The symptoms quickly disappeared when I coincidentally stopped the drug for unrelated reasons. Oh well, but it is a clear illustration of what one of the “best” academic medical centers in the world has to offer a struggling young boy.

Despite the sedation I survived high school and graduated near the top of my class.

I guess the biggest deficit this has left me with is sort of skewing the trajectory of my life. My mother fed into my “being sick” and gained a lot of collateral from it. But worse still, it caused complete neglect of any other possible causes of my problems. My parents in many ways tended to over-interpret every solitary behavior as part of the “disease”. Everything in my life was screened through the filter of this immaterial “disease”. I had enough stacked against me when I was so overwhelmed that they brought me to the psychiatrist in the first place. The neglect of my underlying depression and its being made worse by all the sedating drugs just caused me to just sort of collapse in on myself. And despite being well-liked, I had a difficult time establishing friendships in high school and elsewhere. I had to quit my swim team (when I was 12), something I was amazingly successful at and would have gone far with.

Meanwhile, none of this had the potential to correct itself because of my parents’ own problems. So I have suffered for a long time and have been ostracized from my family.

I just think my case is so typical, because of the path things took, and the fact that I was diagnosed and treated by someone who is rubbing elbows everyday with world leading “experts” on this thing. Clearly, when a disturbed child walks into your office, divalproex, risperidone, and some basic parental psychoeducation, is not going to mean recovery for that child. But yet that’s what their guidelines for “treatment” essentially are.

And to think, there’s a trauma clinic right down the street—where I’ve gotten some treatment—and a stone’s throw away, they’re condemning kids to a diminished life. I’m personally of the belief that the children they’re treating are NOT exceptional in any way, and have problems that could easily be ascribed to factors these people have no interest in considering in a serious manner. If everyone at their clinic presented with classical mania, (edit—the researchers) wouldn’t be famous for anything. So they definitely do not have a clinic full of those kids.

### 2.1. In Response to a Further Question about Effect of the Diagnosis on His Sense of Self and Any Other Side-Effects

I never really believed the label myself like on an intellectual level, because like most young people, I always felt there was a reason for my behavior. I started to put some odd pieces of the puzzle together, like: I have this “disease” and it only manifests itself at home in the presence of 2–3 people that happen to be a part of my life. Then I began to wonder why I had never had another “manic” episode after a few years and realized that adults with the disorder don’t always go years on end without a relapse of that kind of “episode”.

I did however sort of believe it, only because if you tell a kid something long enough, they’ll start to believe it. And of course, if I question my craziness, that’s part of the “illness”. So I got put in a double bind that really did make me feel like I was trapped or going crazy. Many of the arguments with my mother that would land me in hospital began several hours before as an argument solely about wanting to stop my medicines. There is always context.

But the worst part of this, which I have only been recently able to shake within the last year (2008/9), is the defectiveness I felt. Just kind of in some core way. Like I’m totally different. When I was younger, that feeling was a lot stronger and more prominent. Now I feel like a fool for even having given thought after eight years to the question of whether I might go to sleep one night and wake up manic. I decided with my (new) psychiatrist’s support a year ago to stop my medicines. I’m not doing especially well now, but I have at least been able to shake the feelings the diagnosis itself carved into me. The same can’t be said for its physical and social effects though.

I am also gay. And this focus on an immaterial disease brought the issue into my own mind prematurely because of all this psychiatric treatment and it ensured that my family and doctors would completely neglect it (the focus was the “disease”). It made something that isn’t normally a cakewalk something extraordinarily difficult and complicated.

As far as ownership of my behaviors and emotions go, I never believed the diagnosis on an intellectual level and I always knew there were reasons for my behavior, I just couldn’t really recognize them or name them. So I think a question like that would, sadly, be better asked of my parents. How did it affect their perception of everything? It didn’t make me feel not responsible for my actions and on some level I was at least partly sure I wasn’t some defective, degenerating, out-of-control machine.

The mitochondrial disorder thing was a disaster. The testing and consultation dragged out for months. At one point my mom told me they didn’t know if my brain would keep “degenerating”. In effect, “you’re gonna die”. And my psychiatrist was really out to lunch on that one, again. So that experience just profoundly deepened my ignored depression.

I always had terrible sedation from the anticonvulsants and atypical antipsychotics. The sedation from divalproex was unmanageable and had a deadening effect. When I was initially on 7 or 8 drugs, I had terrible tremors, severe memory problems and my head was about as functional as a block of lead.

One very embarrassing problem, which I imagine divalproex is involved with and which my psychiatrist certainly never imagined asking about, was my pubic hair began to fall out. Yep. The amount and frequency that came out was not normal. It was not good.

I also had severe weight gain. From my first contact with these psychotropics, after only 4 months I gained over 50 lbs. I would subsequently lose it when I would stop the medication myself and then gain it back when I was forced back on the medications. This cycle repeated itself 5 times over 8 years. Obviously I couldn’t go back to swimming. Having almost qualified for national swimming championships a year before my diagnosis, I didn’t recognize myself as the cow I was forced to become. This was very troubling. I lost control of my body. After one cycle I gained about 85 lbs in 6 months.

I had sexual dysfunction that would only abate when I stopped the drugs. Every SSRI drug I happened to be put on completely obliterated my sex drive. They were the worst.

I also wonder having never had my prolactin levels tested and having been on risperidone and divalproex for about 7 growing years whether I should get my bone mineral density tested.

I am in psychotherapy, and with a good psychotherapist (finally!). It’s helping a lot.

Sorry for being so long-winded, but that’s the basic extent of things. And I don’t mind you sharing any of it. I read your papers and letters published in the journals, and I have to tell you it gave me a lot of hope and sort of made me feel like the world is a little less crazy.

### 2.2. Further Information

In the face-to-face meeting in 2013, Adam said that his siblings, now all adults, had worked through their issues (partly with therapy) and were reconciled on very good terms. They now had shared insight into the intergenerational patterns of disrupted attachment involving their grandparents and parents. The precipitant to their mother’s investment in Adam’s PBD diagnosis appeared to be a bereavement crisis following the deaths of the maternal grandparents. Adam said he and his siblings were concerned about their mother, who, after Adam left the home, developed a preoccupation with a range of medical complaints and sought out different medical specialists despite normal tests for her alleged medical disorders.

Adam also recalled that early in his treatment he received an SSRI that caused him to have akathisia and agitation with insomnia causing intense frustration—but no core symptoms of mania such as euphoria, flight of ideas or grandiosity. This was diagnosed as “mania”. Afterwards, he never had the reaction to the same extent with further SSRIs. From my inquiries in our 2013 discussion he described how he had never had any core manic symptoms at any point.

If some readers remain skeptical of Adam’s story and his current wellbeing then a mental state examination is worth adding. Across a dinner table over a couple of hours, both I and my psychiatrist colleague (Dr. Anja Kriegeskotten) found ourselves communicating with a very genuine, perfectly sane and intelligent young man with absolutely normal emotional reactivity and good sense of humor. He showed deep insight into the social dynamics of his family and the health system that had engulfed his adolescence. A warm and candid rapport was easily established.

## 3. Discussion

### 3.1. Decontextualized Psychiatry

Adam said: “there was a lot the psychiatrist should have asked about”. Psychiatric symptoms do not occur in a vacuum. In Adam’s words—“there is always context”.

The political history of psychiatry that led to DSM-III in 1980 explains why psychiatric nosology became decontextualized. Broadly speaking psychiatric nosology has been a struggle between two different perspectives, embodied in (1) Emil Kraepelin’s more “medical model” of categorization by symptoms and course of illness, and (2) the “psychobiological” model of Adolph Meyer who advocated that psychiatric interviews should start with a developmental history and the context of the patient’s life. DSM-III adopted a nomothetic, “neo-Kraepelinian” model of diagnosis, based on symptom criteria checklists. This arose out of the need for reliability in diagnosis following an era dominated by psychoanalysis and subjectively inferred psychodynamic conflicts. There was also great geographical variation in the diagnosis of schizophrenia between the USA and Europe that called for more strictly defined diagnostic methodology. But lost was the “Meyerian” ideographic model for diagnosis (partly embodied in DSM-I and DSM-II) that viewed psychiatric syndromes as arising out of individual lives with multiple interactive biopsychosocial causations [8].

Greater reliability of syndrome description does not necessarily mean greater validity of diagnosis. Similar symptomatic presentations can have differing causation in different individuals. The introductions in the DSM-III and DSM-IV manuals specifically warn against reification of diagnoses, and that the DSM must “not be used in a cookbook fashion” [9]. Adam is not alone to suffer from misdiagnosis or diagnosis without consideration of context. The recent publication of DSM-5 occurred amidst controversy. Thousands of mental health clinicians and over 50 mental health organizations signed an online open letter protesting the decontextualized nature of the DSM, the open letter stated:

“… (taxonomic) systems such as this (DSM-5) are based on identifying problems as located within individuals. This misses the relational context of problems and the undeniable social causation of many such problems.”

[10]

Robert Spitzer, head of the APA’s DSM-III committee, that emphasized the nomothetic over the ideographic in psychiatric nosology, recently revised his viewpoint in a foreword to the book *The Loss of Sadness: How Psychiatry Transformed Normal Sorrow into Major Depressive Disorder* [11]:

“(this book) has forced me to rethink my own position. … The very success of the DSM and its descriptive criteria … has allowed psychiatry to ignore basic conceptual issues … especially the question of how to distinguish disorder from normal suffering. … DSM diagnostic criteria … ignored any reference to the context in which they developed.”

Adam and his family had a lot of relational suffering. It may have been beyond the norm for healthier families. But the suffering was embedded in intergenerational family dynamics. Now in their mid to late twenties Adam and his siblings have insight into these dynamics. That insight has been liberating for them.

### 3.2. Pediatric Bipolar Disorder—Emblematic Diagnosis for Decontextualization

The head of the DSM-IV committee, Allen Frances, has criticized aspects of DSM-5. He also criticized PBD [12]. Although Frances noted that strict adherence to DSM-IV criteria would’ve ruled out PBD, the nomothetic and by default biomedical model of DSM-IV allowed PBD to flourish within the Bipolar Disorder—Not Otherwise Specified (BD-NOS) category. A recent article [13] goes further to criticize the nomothetic medical model:

“A classic criticism against medicalization applies: the “medical gaze” locates the problem and the place of treatment within the individual child, and neglects possible social dimensions of the problem.”

Several factors appear to have fueled the PBD epidemic: The pharmaceutical industry’s influence on research, medical education and consumer groups; a desire for a blame-free biological explanation to distressing family problems; a human individual and societal need to repress trauma; and diagnostic upcoding in the U.S. health system that rations treatment according to DSM diagnoses [14]. To this could be added academic hubris: Adam noted that by defining a “new” disorder, the academic child psychiatric center that he attended gained a degree of fame.

The PBD academic literature is grossly lacking in research into contextual factors. A systematic review [15] of over a thousand PBD articles for terms such as attachment theory, maltreatment and child neglect found these terms to be almost completely absent. PTSD, trauma and child abuse terms were infrequently referred to and generally only in passing. Rates of physical abuse and sexual abuse in cohorts of research subjects from the two academic child psychiatric centers that pioneered PBD were far below rates in community surveys and emotional abuse appears to have not been considered at all. The methodology in PBD research leans heavily on structured parent interviews. As in research, so in clinical practice. As Adam informed me, the sessions with his psychiatrist involved his mother and the psychiatrist discussing his symptoms and little space for he to ever talk about the physical and emotional abuse by his brother, or the background to the conflict with his mother.

DSM-5 has introduced DMDD with the primary rationale to curb the diagnosis of PBD. However DMDD still embodies the same decontextualized model. A similar systematic literature review of 76 articles found minimal mention of attachment, maltreatment and parenting and family dynamic factors [16]. It seems possible that without recognition of context, a child could go through a similar experience to Adam with a DMDD label. In contrast, another diagnosis submitted for inclusion in DSM-5, Developmental Trauma Disorder (DTD) [17], embedded contextual factors in its criteria. The DSM-5 committee rejected DTD mainly on the basis that symptoms overlapped with other disorders, even though the same critique has been leveled at DMDD [18]. It appears that many researchers prefer to count symptoms rather than explore where they come from.

### 3.3. Over-Medicating and Side-Effects

Adam described a staggering amount of psychotropic polypharmacy with a litany of side-effects. The treatment Adam received could trigger Medical Board investigation in Australia, yet Adam informed me his legal inquiries indicated his treatment would be deemed “standard practice” where he lived. Nonetheless there is increasing criticism of these medication practices with reports of iatrogenic morbidity and mortality in the U.S. media [19] and academic literature [20]. A health system that forces many child psychiatrists into brief “med checks” is seen as a serious problem. An op-ed in the *Los Angeles Times* by A/Prof Laurel Williams expounds on these problems [21].

Adam had an akathisia/agitation reaction to an SSRI at age 12. These are now well described in the literature [22,23]. However in the 1990s there was dispute about such reactions, and pharmaceutical manufacturers tended to deny the existence of SSRI induced agitation. I recall seeing several adolescents develop the reaction when I worked on a mood disorders unit for young people in the mid-1990s. At the time I prescribed SSRIs liberally. We now know that at least some published SSRI drug trials suppressed data about these reactions [24]. Patients like Adam suffered if their treating psychiatrists were kept in the dark about side-effects by the academic literature. For example, I recall prescribing quetiapine to help patients on antipsychotics lose weight—on the basis of fraudulent studies sponsored by AstraZeneca (London, UK), the manufacturer of quetiapine (Seroquel) [25].

### 3.4. The Iatrogenic Harm of Erroneous Labeling

Adam eloquently describes the impact of the diagnosis upon his sense of identity and familial relationships. The central task of adolescence is individuation [26]. Identity development can be severely damaged by a misdiagnosis of PBD, where one’s every thought and feeling can be doubted as whether it is a part of self or, as Adam says, some “immaterial disease”. As Adam also indicates, the impact of sedating medications on subjective experience adds to the impairment. Despite his success at university and the psychotherapy that has helped him work through his family conflicts, he still feels a disturbing lack of connection with his sedated adolescent years.

This damage to identity formation in children with PBD diagnoses has been noted [27,28]. Even where biomedical explanations may be warranted, there is evidence that a biomedical explanation is likely to foster greater rather than less stigma and induce “prognostic pessimism” [29]. Adam is at the crest of a tsunami of thousands who’ve grown up with the PBD diagnosis. Many of these young adults do not have the resources Adam has marshaled. It is an area that demands further research. With PBD and other diagnoses psychiatrists are often faced with having to “undiagnose” patients, and given the entanglement of label with identity the task of “undiagnosing” requires tact and much support [30].

### 3.5. Projective Identification and “Munchausen’s by Proxy”

It is traditional wisdom in child psychiatry that parents often project unresolved issues onto their offspring. The children may identify and act out accordingly. Some extreme versions of this can lead to “Munchausen’s syndrome by proxy”, where a parent, through having an ill child, vicariously gains desired attention from respected medical experts for unmet and disavowed dependency needs. It appears that once Adam left the home his mother produced spurious medical symptoms and diagnoses for herself, in other words her own likely case of Munchausen’s disorder.

An early critique of PBD [31] noted that not only could parents have a psychological investment in the PBD diagnosis, but so too could a range of others including the pharmaceutical industry, academic child psychiatry, schools and consumer advocacy groups. The authors speculated whether PBD may be a “variant on Munchausen’s syndrome”.

This is not to say that there need be any negligence or mal-intent at all. Factors operate at systemic and subconscious levels. Adam’s mother, his doctors and others no doubt acted with Adam’s best interests in mind. A dominant paradigm is hard to see when you’re living and working within it.

### 3.6. A Paradigm Problem in Psychiatry

Silove [1] (1990) in his prophetic article on psychiatric trends in North America, referenced both the eminent U.S. child psychiatrist Eisenberg [32] and a president of the Canadian Psychiatric Association, Lipowski [33], both of whom used the terms “brainless psychiatry” and “mindless psychiatry”. The mid 20th century hegemony of Freudian psychoanalysis tended at its extreme to be a “brainless” model that Eisenberg and Lipowski were highly critical of. But the thrust of their late 1980s warnings concerned the rise of “mindless” psychiatry, or, as Silove called it, “biologism”.

What is it but “biologism” that influenced Adam’s psychiatrist and other doctors to misconstrue parent-child conflict as mania, prescribe him so much medication and misdiagnose polypharmacy side-effects as a neurological disorder involving months of high-tech investigations?

In addition to being a method of inquiry, science is a social process and there is a vast research literature concerning the sociology of science. Scientific disciplines do not build on knowledge in a purely linear fashion, but at times undergo dramatic upheavals according to paradigm shifts [34]. The dominant paradigm governs what is acceptable to study, research, publish and practice. Softer sciences like psychiatry can be more susceptible to extreme paradigm shifts. The history of psychiatry reflects this. The issue is not simply an academic one (pun intended). What is emphasized in teaching and research plays out in practice—with real life consequences, as Adam well describes.

### 3.7. Training in Psychiatry

Silove [1] described a narrowing of psychiatric training by 1990 in the USA:

“In the area of teaching, North American clinicians schooled in more comprehensive clinical traditions of yesteryear, express fears that training programmes in psychiatry offer little more than instruction in matching formula-based “diagnoses” to specified pharmacological treatments.”

Silove was hopeful Australasian psychiatry’s grounding in the “eclectic” biopsychosocial model could buffer it from biologism. In the years since Silove’s warning, Australian and New Zealand psychiatrists in training have still had to pass written case histories, including long-term psychotherapy cases. The oral viva exam still incorporates “long cases” with real life patients. The presentation of a diagnostic case formulation in these exams—a narrative of the patient’s psychopathology within the developmental biopsychosocial context—is still more valued by the RANZCP examiners, as I know from my time as a case histories examiner, than symptom criteria-based diagnoses such as in DSM-5 or ICD-10.

Nonetheless biologism in psychiatry is a global issue. Boyce [35], in a presidential address to the RANZCP annual congress titled “Restoring Wisdom to the Practice of Psychiatry”, noted in Australia and New Zealand there had also been a:

“… dumbing down” of psychiatry (due to) “increased service demand, the deification of DSM, the influence of the pharmaceutical industry, a misunderstanding of evidence-based medicine, managerialism and the influence of consumerism.”

However unlike Australasia where the focus is still generally on clinical need, the U.S. health insurance industry rations treatment according to DSM diagnoses and U.S. academic psychiatry and education has been more dependent on pharmaceutical funding than in Australasia.

On my recent study trip to the USA I was privileged to visit some centers of excellent holistic psychiatric training, but these may not reflect the norm. At the 2013 APA annual meeting in San Francisco, a psychiatric resident told me how his group had been practicing for their board exams. Their experienced tutor asked for the “diagnostic formulation” for the patient who was interviewed, but none of the residents had heard of a “formulation” in their entire psychiatric training. I was also informed that the U.S. National Board of Medical Specialties (NBMS) exams were going to be devoid of real life patients, using written clinical vignettes in future.

Of U.S. psychiatry training, Tasman [36] wrote:

“Many fear that we are in danger of training a generation of psychiatrists and physicians who lack basic psychotherapeutic skills or a framework for understanding mental functioning from a psychodynamic perspective.”

The loss of the biopsychosocial diagnostic formulation compounds the demise of psychodynamic theory in psychiatric training. In practice this means that the patient’s inner life is devalued or ignored, surface symptoms are taken at face value and underlying causation and meanings may remain unexplored. This could explain why a highly qualified psychiatrist with strong academic credentials and with the best intentions, could, as Adam describes, fail to explore his inner thoughts and feelings and the family context.

## 4. Conclusions

Psychiatry needs a paradigm shift to one that is neither “brainless” nor mindless”. Bracken *et al.* [37] described the dominant paradigm in psychiatry as a “technological paradigm” that has relegated relationships, meanings and values to secondary concerns and focused on symptomatology and interventions “independent of context”. They argued psychiatry must break free from the constraints of this technological paradigm:

“Psychiatry is not neurology, it is not a medicine of the brain. Although mental health problems undoubtedly have a biological dimension, in their very nature they reach beyond the brain to involve social, cultural and psychological dimensions. These cannot always be grasped through the epistemology of biomedicine.”

It should be obvious actually.

Stepping out into the San Franciscan sunshine at the 2013 APA conference, I was greeted by several hundred protestors chanting in loud unison: “APA, APA, how many kids did you drug today?” The protestors were from the Scientology backed Citizens Commission for Human Rights (CCHR). Whilst I did not entertain joining them—I am a psychotropic prescriber after all—I couldn’t help but ponder the question that echoed around the surrounding skyscrapers.

I heard that Prof. Joel Paris, editor-in-chief of the *Canadian Journal of Psychiatry* stated in a presentation at the 2012 APA annual meeting:

“When psychiatrists 50 years from now look back on our current era in psychiatry, they will understand that the diagnosis of pediatric bipolar disorder is the greatest scandal to ever befall psychiatry.” Prof. Paris confirmed: “This is exactly what I said.”

—Personal Communication [38]

What Adam went through was scandalous, even if well-meaning. But his story demands action now and shouldn’t have to wait for the verdict of history. He is at the crest of a tsunami of young people who have been affected by the PBD diagnosis. Others are starting to voice their stories as in documentaries like “Letters from Generation Rx” [39]. Their stories need to be heard. Psychiatry needs to be grounded in listening to our patients. By listening to their full stories and by understanding the full context of whatever problems are brought forth, we may offer more tailored beneficial assistance across the biopsychosocial spectrum, and, at the very least, do no harm.
